# A Functional Signature in the Developing Cerebellum: Evidence From a Preclinical Model of Autism

**DOI:** 10.3389/fcell.2021.727079

**Published:** 2021-09-03

**Authors:** María Berenice Soria-Ortiz, Pamela Reyes-Ortega, Ataúlfo Martínez-Torres, Daniel Reyes-Haro

**Affiliations:** Departamento de Neurobiología Celular y Molecular, Instituto de Neurobiología, Universidad Nacional Autónoma de México—Campus Juriquilla, Querétaro, Mexico

**Keywords:** astrocytes, internal granular layer, autism spectrum disorder, calcium wave, glial fibrillary acidic protein, valproate

## Abstract

Autism spectrum disorders (ASD) are pervasive neurodevelopmental conditions detected during childhood when delayed language onset and social deficits are observed. Children diagnosed with ASD frequently display sensorimotor deficits associated with the cerebellum, suggesting a dysfunction of synaptic circuits. Astroglia are part of the tripartite synapses and *postmortem* studies reported an increased expression of the glial fibrillary acidic protein (GFAP) in the cerebellum of ASD patients. Astroglia respond to neuronal activity with calcium transients that propagate to neighboring cells, resulting in a functional response known as a calcium wave. This form of intercellular signaling is implicated in proliferation, migration, and differentiation of neural precursors. Prenatal exposure to valproate (VPA) is a preclinical model of ASD in which premature migration and excess of apoptosis occur in the internal granular layer (IGL) of the cerebellum during the early postnatal period. In this study we tested calcium wave propagation in the IGL of mice prenatally exposed to VPA. Sensorimotor deficits were observed and IGL depolarization evoked a calcium wave with astrocyte recruitment. The calcium wave propagation, initial cell recruitment, and mean amplitude of the calcium transients increased significantly in VPA-exposed mice compared to the control group. Astrocyte recruitment was significantly increased in the VPA model, but the mean amplitude of the calcium transients was unchanged. Western blot and histological studies revealed an increased expression of GFAP, higher astroglial density and augmented morphological complexity. We conclude that the functional signature of the IGL is remarkably augmented in the preclinical model of autism.

## Introduction

Autism Spectrum Disorder (ASD) refers to a group of neurodevelopmental disorders characterized by social impairment, communication deficits, stereotypies, and repetitive behaviors. ASD is diagnosed by the age of two and the prevalence is about 1% of the global population, but little is known about the neurobiology of the disorder, particularly before diagnosis. Motor disorders associated to the cerebellum are frequently observed in patients diagnosed with ASD before the onset of language or social deficits (Lloyd et al., 2013; Mcphillips et al., 2014; Mosconi et al., 2015). The cerebellum is a site of ASD gene-associated co-expression during early postnatal development, especially in the granular layer (Menashe et al., 2013; Wang et al., 2014). Granule cells (GCs) of the cerebellum represent nearly half of the neurons of the rodent or primate brain and integrate multiple sensory modalities (Wang et al., 2014). GC axons give rise to parallel fibers and synapse onto Purkinje cells (PCs). Climbing fibers that project from the inferior olivary nucleus to PCs can drive the plasticity of the resulting signal, since there is a sole inhibitory output from PCs into the deep cerebellar nuclei which in turn project to the thalamus and other brain regions (Huang et al., 2013; Wang et al., 2014). PC loss (Skefos et al., 2014) and the increased expression of astroglial markers, such as aquaporin-4, connexin 43 and GFAP have been reported in *postmortem* studies of ASD patients (Laurence and Fatemi, 2005; Fatemi et al., 2008; Edmonson et al., 2014). Neuron-glia communication is required for normal functioning of the brain during early neurodevelopment and through life. Astroglia are part of the tripartite synapses and respond to neuronal activity with calcium transients that propagate to neighboring cells, a signaling mode known as a calcium wave (Schipke and Kettenmann, 2004; Perea and Araque, 2005). This form of intercellular communication is implicated in the proliferation, migration and differentiation of neural precursor cells (Weissman et al., 2004). Bioinformatic studies have shown that ASD gene-associated co-expression networks are highly expressed in the cerebellar granule layer and that genes involved in calcium signaling are proposed to be dysregulated in the developing brain of autistic children (Menashe et al., 2013; Zeidán-Chuliá et al., 2013). However, it is unknown whether neuron-glia communication associated with calcium signaling is disturbed in the early neurodevelopment of the autistic brain. Murine models of ASD open the possibility to investigate this problem in a developmental window prior to diagnosis. Prenatal valproate (VPA) exposure is a commonly used preclinical model of ASD to explore mechanistic and therapeutic investigations (Nicolini and Fahnestock, 2018; Varman et al., 2018; Wang et al., 2018). VPA is a mood stabilizer and anticonvulsant drug, but prenatal administration in humans results in linguistic, motor and cognitive deficits (Moore et al., 2000). The VPA model reproduce these deficits including reduced dendritic arborization of PCs, delayed GC precursor migration and neuronal apoptosis (Rodier et al., 1997; Ingram et al., 2000; Varman et al., 2018; Wang et al., 2018). Thus, the aim of this study was to test depolarization of IGL on cellular responses associated with calcium signaling in mice prenatally exposed to VPA.

## Methods

### Animals

Mice were handled according to the National Institute of Health’s Guide for the Care and Use of Laboratory Animals and the Institutional Committee on Animal Care and Use of Laboratory Animals of the Institute of Neurobiology, UNAM. Briefly, CD-1 or GFAP-eGFP transgenic mice (Nolte et al., 2001) were mated and pregnancy was confirmed by a vaginal plug corresponding to embryonic day 0 (E0). The pregnant mice were housed individually under a 12 h/12 h light/dark cycle with controlled temperature, and food and water *ad libitum.* A single intraperitoneal injection (IP) of sterilized saline solution (0.9%) or VPA (Sigma-Aldrich, St. Louis, MO, United States) was administered at E12.5 (Varman et al., 2018). VPA was dissolved in sterilized saline solution (0.9%) as follows: 500 mg/Kg for CD-1 and 300 mg/Kg for GFAP-eGFP dams. Only male pups were used for this study based on ASD incidence (4:1) (Kim et al., 2013; Perez-Pouchoulen et al., 2016; Melancia et al., 2018). For behavioral and histological experiments, the sample size (*n*) represents the number of animals used while *N* represents the number of litters. On the other hand, for calcium imaging experiments, *n* represents the number of slices, while *N* is the number of mice.

### Behavioral Testing

The latency to reach the nest and righting reflex were selected as prognostic behavioral tools to identify subjects with sensorimotor deficits during early postnatal development.

#### Latency to Reach the Nest

The nest-seeking test was carried out at P8 for both CTL and VPA experimental groups (n_CTL_ = 35 from 10 litters, n_VPA_ = 47 from 13 litters). The pups were placed in the center of a 35 × 20 cm^2^ plastic cage containing clean bedding and home bedding in opposite sides (∼5 cm width) with 25 cm of. Olfactory cues were prevented by cleaning the separation cage after each trial. The latency to reach the nest was recorded immediately after the mouse’s head touched the home bedding (Schneider and Przewłocki, 2005; Roullet et al., 2010; Varman et al., 2018).

#### Righting Reflex

The righting reflex is a mouse pup’s motor ability to flip onto its feet from supine position. At P9, the pups were placed on their backs on a flat surface and held in that position for 5 s. Next, they were released and the time it took them to return to prone position was recorded for the CTL (n_CTL_ = 16 from 4 litters) and the VPA (n_VPA_ = 12 from 3 litters) experimental groups (Feather-Schussler and Ferguson, 2016; Kazlauskas et al., 2016).

### Western Blot Analysis

Cerebella from CD-1 male pups (P8) were dissected from 4 litters, for both the CTL and VPA groups. Briefly, four sets of cerebella for each experimental group (4 cerebella per set, *n* = 16) were homogenized in iced-cold glycine lysis buffer (in mM: 200 Glycine, 150 NaCl, 50 EGTA, 50 EDTA, 300 sucrose, pH 9.0) and protease inhibitor (Sigma-Aldrich, St. Louis, MO, United States), followed by protein isolation and quantification with a Bradford assay (Bio-Rad, Hercules, CA, United States) (Bradford, 1976). An equal amount of protein (10 μg) per lane was resolved in a 10% polyacrylamide gel. The proteins were transferred to PVDF membranes, blocked with 5% non-fat dry milk in Tris-buffered saline (TBS), 0.1% Tween 20 (TBS-T) for 3 h at room temperature. The membranes were incubated overnight at 4°C with the primary antibody goat polyclonal anti-GFAP 1:1,000 (Santa Cruz, Dallas TX, United States) or rabbit anti-Actin 1:1,000 (Santa Cruz, Dallas TX, United States). The membranes were rinsed three times (15 min/each) with TBS-T and primary antibodies were detected after incubation (3 h) with either rabbit anti-goat IgG-AP (1:2,000) or goat anti-rabbit IgG-AP (1:2,000) (Santa Cruz, Dallas, TX, United States). Alkaline phosphatase activity was detected with BCIP/NBT AP-conjugate substrate reaction kit (Bio-Rad, Hercules, CA, United States) after post-secondary washes with TBS-T. The images of the Western blot bands were acquired with the Image based Gel Doc^TM^ EZ Gel Documentation System (Bio-Rad, Hercules, CA, United States). Optical density was calculated with Image Lab 3.0 software (Bio-Rad, Hercules, CA, United States) and normalized with the β-Actin bands.

### Histology

Histological studies were performed in GFAP-eGFP transgenic mice at P8 (n_CTL_ = 3, n_VPA_ = 4, 3 litters each group). Briefly, mice were deeply anesthetized with an IP injection (100 mg/Kg) of pentobarbital and intracardially perfused with chilled (4°C) paraformaldehyde (PFA 4%) in 0.1 M phosphate-buffered saline (PBS, pH 7.4) as previously described (Varman et al., 2018). Brains were carefully isolated and kept in PFA for another 24 h, then washed and cryoprotected in 30% of sucrose at 4°C. Brains were frozen in polyvinyl alcohol-polyethylene glycol medium (Tissue-Plus, Fisher Health Care) at −80°C and horizontal sections (35 μm) including Crus I/II, were obtained with a cryostat (Leica CM1850). Sections were stored in cryoprotectant solution (30% ethylene glycol-30% sucrose in PBS) at −20°C. The histological sections were washed three times (10 min/each) with PBS, counterstained with 4′, 6-diamidino-2-phenylindole (DAPI, 1:16,000) and mounted with Vecta-Shield (H1000, Vector Laboratories, Burlingame, CA, United States).

### Cell Counting

The GFAP-eGFP transgenic mouse line facilitated the identification of the IGL. Bergmann glia and cerebellar white matter set the boundaries of the IGL and visual fields of 50 × 200 μm were selected for the analysis. Briefly, three images per slice from the internal granular layer (IGL) of the Crus I/II regions (n_CTL_ = 3, n_VPA_ = 4, in triplicated samples) were acquired with an ApoTome microscope and a Zeiss LSM 780 confocal microscope (Zeiss, Germany) using Zen2012 Blue Edition software and processed in ImageJ. Cell somas were counted automatically in CellProfiler 2.1.1 software.

### Morphological Analysis

Three Z-stack images per slice from the IGL of the Crus I/II regions (*n* = 4 each experimental group, in triplicated samples) were acquired with a Zeiss LSM 780 confocal microscope (Zeiss, Germany) using an amplification of 63x. Images were analyzed with the MATLAB-based script 3DMorph (York et al., 2018). Individual cells (Cells_CTL_ = 426 and Cells_VPA_ = 445) were processed into a 3D skeleton configuration, keeping all branches. Cell volume, number of branchpoints, and branch length of each cell were estimated. Sample size (n) represents the average of the number of cells per animal.

### Sulforhodamine B Staining of Astrocytes

Mice were intraperitoneally injected with sulforhodamine B (SRB; 20 mg/Kg) 4 h prior the brain slice sectioning. This approach was previously reported for astrocyte staining (Nimmerjahn et al., 2004; Appaix et al., 2012). We further corroborated the selectivity of SRB for astrocytes in the GFAP-eGFP transgenic mouse line. Our results showed that 84 ± 2% of GFAP-eGFP^+^ cells incorporated SRB in the IGL of Crus I/II regions (*n* = 12, *N* = 3). We conclude that SRB stains mainly astrocytes (see Supplementary Figure 1).

### Acute Brain Slice Preparation and Calcium Imaging

Brain slices were obtained as previously described (Reyes-Haro et al., 2010, 2013; Labrada-Moncada et al., 2020). Briefly, acute cerebellar slices were prepared from 8- to 10-day-old (P8-P10) CD-1 or GFAP-eGFP mice (Nolte et al., 2001). After decapitation, the brain was immediately removed and coronal slices (250 μm) containing Crus I/II regions were obtained with a vibratome (VT1000s, Leica) and transferred to ice-cold oxygenated artificial cerebrospinal fluid (aCSF, in mM: 134 NaCl, 2.5 KCl, 2 CaCl_2_, 1.3 MgCl_2_, 26 NaHCO_3_, 1.25 K_2_HPO_4_, 10 glucose, pH = 7.4). The slices were obtained and stored at room temperature in oxygenated aCSF for at least 30 min, followed by incubation with the Ca^2+^ indicator dye Fluo-4 AM (10 μM, AAT Bioquest, Sunnyvale, CA, United States) for another 30–45 min at 37°C. The slices were washed with aCSF for 30 min, transferred to the recording chamber and perfused with oxygenated aCSF (2 ml/min) at room temperature (20–22°C). Calcium imaging experiments were performed under a cooled camera (SensiCam; PCO.Edge 4.2, Kelheim, Germany) coupled to an Olympus upright microscope (BX51WI, Miami, FL, United States) and a LED module (X-Cite X-LED1 lumen Dynamics Fremont, CA, United States; BDX: 450–495 nm for Fluo-4AM and GYX: 540–600 nm for SRB). The calcium waves were evoked by depolarization (20 pulses of 200 μA at 10 Hz evoked with a DS3 Isolated Current Stimulator, Digitimer Ltd., Fort Lauderdale, FL, United States) with a pipette pulled from thin-walled borosilicate glass (outer diameter 1.5 mm, inner diameter 0.87 mm) with a P97 puller (Sutter Instruments, Novato, CA, United States). The stimulation electrode (with a tip opening of ∼20 μm) was filled with aCSF and placed on top of the slice, gently touching the upper cells of the IGL. Then, the slice was allowed to recover from mechanical stress for at least 5 min. Image analyses and processing were performed with ImageJ/FIJI software as previously described (Haas et al., 2006; Reyes-Haro et al., 2010; Labrada-Moncada et al., 2020). The image acquisition protocol consisted of 200 s at 1 Hz. The first 15 s were defined as the pre-stimulus window before depolarization and the average of the corresponding images represented the basal fluorescence F_b_. The threshold of the evoked intracellular calcium transients ([Ca^2+^]_i_) was estimated as Δ*F* = Δ*F/F_b_*, where Δ*F* is the relative change of the fluorescence over the basal fluorescence *F*_b_. The area under the curve was estimated with an integration method based on the trapezoidal rule. The maximum length and number of recruited cells within the calcium wave were estimated using concentric rings with 50 μm increments around the site of stimulation. The speed of propagation was defined as (maximum length)/(t_1_−t_0_); where the t_0_ was the time when the first cells were activated immediately after the stimulus and t_1_ was the time when the farthest cells were activated.

### Statistical Analysis

Statistical analysis was performed using Origin Lab 8.0 software (Origin Laboratories, Northampton, MA, United States) and includes the Shapiro-Wilk test to determine a normal distribution, t-Student or Mann-Whitney tests to analyze differences between CTL and VPA groups in populations with normal and non-normal distributions. Data are reported as mean ± SEM. *P*-values < 0.05 were considered significant.

## Results

### Sensorimotor Deficits in the VPA Model

**FIGURE 1:**
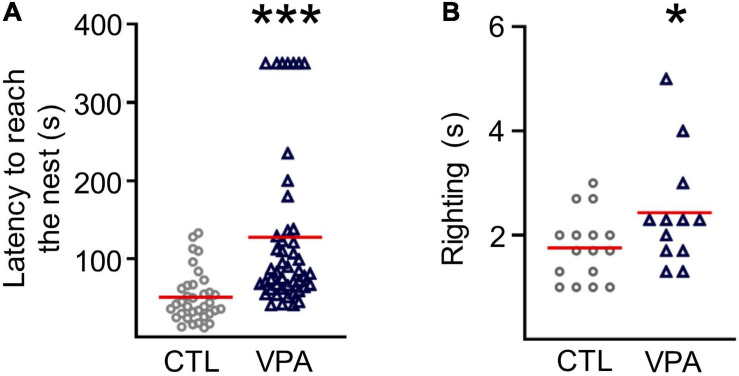
Sensorimotor deficits in the VPA model of autism. **(A)** Graph of latency to reach the nest performed at P8 (n_CTL_ = 35, N_CTL_ = 10; n_VPA_ = 47, N_VPA_ = 13). **(B)** Graph of the righting reflex latency performed at P9 (n_CTL_ = 16, n_VPA_ = 12). Statistical test used were Shapiro-Wilk, Mann-Whitney. Data are presented as mean ± SEM. *p < 0.05, ***p < 0.0001. CTL, control; VPA, valproic acid.

In this work, we tested prenatal exposure to VPA on sensorimotor performance through postnatal development (Figure 1). The latency to reach the nest increased in VPA-treated pups (127.7 ± 14.9 s, n_VPA_ = 47, N_VPA_ = 13, *p* = 3 × 10^–7^) when compared to the CTL group (51.2 ± 5.5 s, n_CTL_ = 35, N_CTL_ = 10; Figure 1A). The righting reflex latency was augmented in VPA-treated pups at P9 (2.43 ± 0.3 s, n_VPA_ = 12, N_VPA_ = 3, *p* = 0.05) when compared to CTL group (1.75 ± 0.16 s, n_CTL_ = 16, N_CTL_ = 4; Figure 1B).

### The Functional Signature of IGL Is Increased in the VPA Model

**FIGURE 2:**
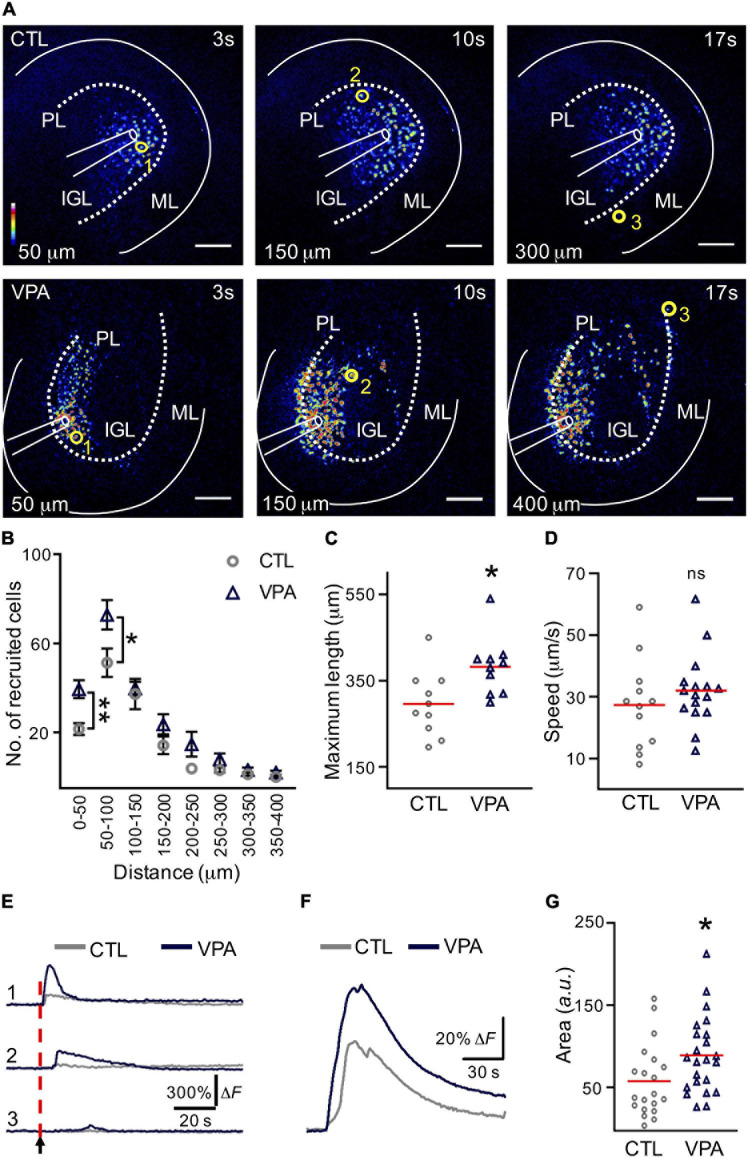
The calcium wave is augmented in the VPA model. **(A)** Evoked calcium waves in control (CTL) and VPA groups. Pseudocolors (from blue to white) represent the intensity of the fluorescent calcium indicator (Fluo4-AM). Scale bar 100 μm. **(B)** Graph of the recruited cells per distance (n_CTL_ = 10, N_CTL_ = 7, n_VPA_ = 10, N_VPA_ = 9). **(C)** Maximum length of the calcium wave (n_CTL_ = 10, N_CTL_ = 7, n_VPA_ = 10, N_VPA_ = 9). **(D)** Calcium-wave speed (n_CTL_ = 12, N_CTL_ = 8, n_VPA_ = 16, N_VPA_ = 12). **(E)** Examples of the activation of three cells recruited at different distances in the evoked calcium wave [yellow circles in **(A)**: cell 1, 50 μm; cell 2, 150 μm; cell 3, 300 or 400 μm]. **(F)** The mean amplitude of the calcium transients was estimated from all the recruited cells within the calcium wave in both CTL (Cells_CTL_ = 1222, n_CTL_ = 20, N_CTL_ = 13) and VPA (Cells_VPA_ = 967, n_VPA_ = 23, N_VPA_ = 15) experimental groups. **(G)** Summary of the mean of amplitudes of the calcium transients obtained from all the recruited cells from CTL and VPA groups. IGL, internal granular layer; PL, Purkinje layer; WM, white matter. Data analyzed by Shapiro–Wilk, Mann–Whitney U, *p < 0.05. Values are mean ± SEM.

The VPA-treated mice with autistic-like sensorimotor deficits were selected and acute slices containing the Crus I/Crus II regions of the cerebellum were depolarized at the IGL to evoke a calcium wave (Figure 2A). The number of recruited cells increased significantly (+82%) within the initial distance; 0–50 μm (CTL: 22 ± 3 cells, VPA: 40 ± 4 cells, *p* < 0.001). The second distance (50–100 μm) showed a similar increase (+43%; CTL: 51 ± 6 cells, VPA: 73 ± 7 cells, *p* < 0.02), while no significant differences were found for the remaining distances (Figure 2B and Supplementary Table 1). The maximum length of the calcium wave propagation was increased (+29%) in VPA-treated mice (382 ± 22 μm, n_VPA_ = 10, N_VPA_ = 9, *p* = 0.01) when compared to the CTL group (295.9 ± 24 μm, n_CTL_ = 10, N_CTL_ = 7) (Figure 2C). The velocity of the calcium wave propagation showed no differences between CTL (27.3 ± 4 μm/s, n_CTL_ = 12, N_CTL_ = 8) and VPA (32 ± 3 μm/s, n_VPA_ = 16, N_VPA_ = 12, *p* = 0.3) groups (Figure 2D). The calcium transients decreased proportionally to the distance from the origin of the depolarization (Figure 2E). The mean amplitude of the calcium transients increased +53% (Cells_CTL_ = 1,222, n_CTL_ = 20, N_CTL_ = 13, Cells_VPA_ = 967, n_VPA_ = 23, N_VPA_ = 15) for the VPA group (89 ± 10 *a. u.*, *p* = 0.01) when compared to the CTL group (58 ± 10 *a. u.*) (Figures 2F,G). Based on these results we conclude that the calcium wave recruited more cells, reached longer distances and increased the mean amplitude of the calcium transients in the VPA model.

### Astrocyte Recruitment Within the Calcium Wave Is Augmented in the VPA Model

**FIGURE 3:**
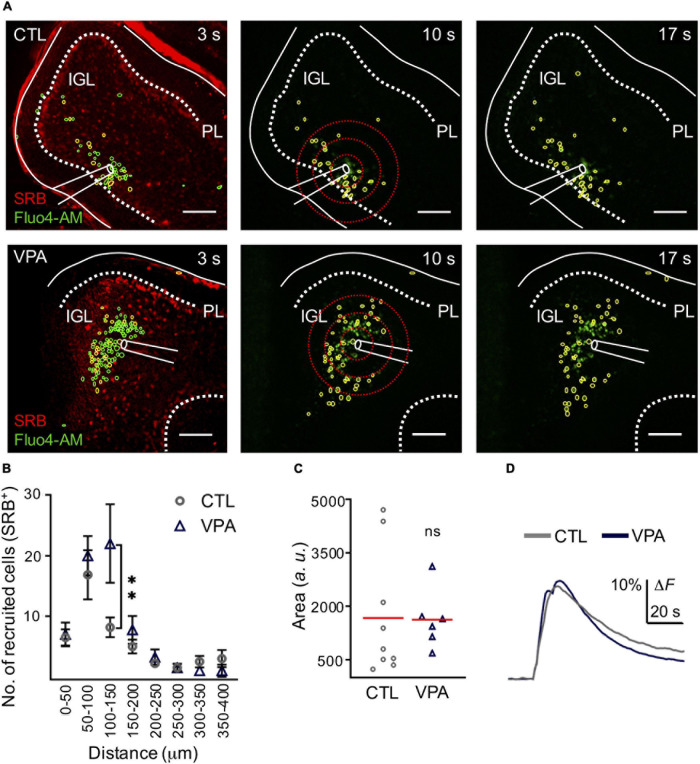
Astrocyte recruitment is augmented within the calcium wave in the VPA model. **(A)** Representative CTL and VPA images of an evoked calcium wave (Fluo4-AM in green) in IGL; at three different times (3, 10, and 17 s). Regions of Interest (ROIs) showed a fraction of Fluo-4AM cells that incorporated SRB (yellow ROIs), the rest of the recruited cells (green ROIs). Scale bar 100 μm. **(B)** Histogram of SRB^+^ cell recruitment per distance (n_CTL_ = 6, n_VPA_ = 4, and N_CTL_ = 3 and N_VPA_ = 3). **(C)** The mean amplitude of the calcium transient was estimated from all SRB^+^ recruited cells within the calcium wave in both CTL (Cells_CTL_ = 299, n_CTL_ = 9, N_CTL_ = 4) and VPA (Cells_VPA_ = 340, n_VPA_ = 6, N_VPA_ = 3) experimental groups. **(D)** Summary of the mean amplitude of the calcium transient obtained from all the recruited cells from CTL and VPA groups. IGL, internal granular layer; PL, Purkinje layer. Data analyzed by Shapiro–Wilk, and Student’s *t*-test, **p < 0.05. Values are mean ± SEM.

We used SRB, a fluorescent dye that is preferentially incorporated by astrocytes (>80% GFAP^+^ cells, *n* = 12, *N* = 3) and observed that the evoked calcium wave recruited SRB^+^ cells (>50%; *n* = 8, *N* = 5) in the IGL (Supplementary Figure 1). Our next step was to test astrocyte recruitment and the corresponding mean amplitude of the evoked calcium transient in the VPA model. Our results showed that SRB^+^ cell recruitment was significantly augmented (+168%) within 100–150 μm ratio (CTL: 8 ± 1.6 SRB^+^ cells, VPA: 22 ± 6.5 cells, *p* < 0.03); but the mean amplitude of the evoked calcium transient was 1,671 ± 578 *a. u.* and 1,629 ± 6.336 *a. u.*, for the CTL (Cells-_CTL_ = 299, n_CTL_ = 6, N_CTL_ = 4) and VPA (Cells-_VPA_ = 340, n_VPA_ = 4, N_VPA_ = 3), respectively (*p* < 0.4) (Figure 3 and Supplementary Table 2). Based on these results we conclude that the evoked calcium wave augmented astrocyte recruitment without changes in the mean amplitude of the calcium transient, in the VPA model.

### GFAP Expression, Astroglial Density and Morphological Complexity Are Increased in the VPA Model

**FIGURE 4:**
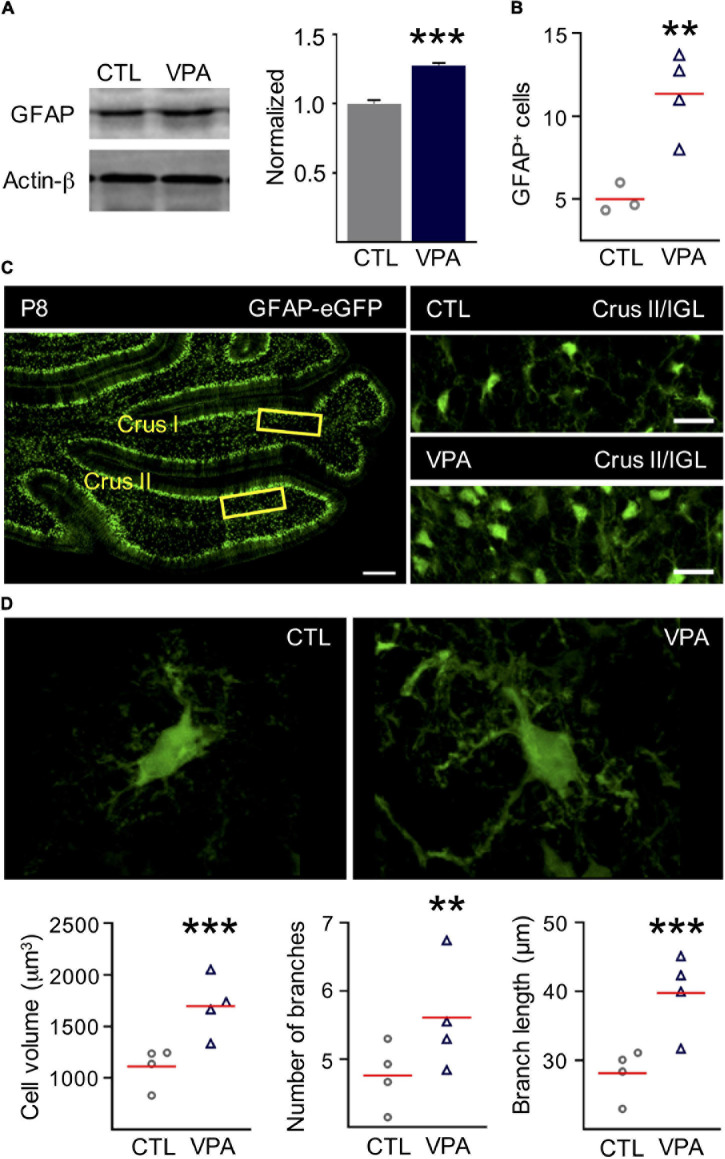
GFAP expression, astrocyte density, and morphology complexity in the IGL are increased in the preclinical model of autism. **(A)** Representative Western blot showing the expression of GFAP protein (50 KDa) for CTL and VPA experimental groups. Actin-β (43 KDa) was used as internal control. The band intensities were normalized for CTL (*n* = 16) and VPA (*n* = 16) experimental groups **(B,C)**. The density of GFAP^+^ cells is increased in the VPA model. Cell counting was performed in the cerebellar regions Crus I and Crus II of GFAP-eGFP transgenic mice (n_CTL_ = 3, n_VPA_ = 4). **(C)** Left, the horizontal section from the cerebellum shows Crus I and Crus II regions and the internal granular layer (IGL, yellow rectangles). Scale bar 100 μm. Right, representative sections of IGL in Crus II showing GFAP^+^ cell density CTL and VPA experimental groups. Scale bar 20 μm. **(D)** Representative images and analysis of the astrocytic morphology showing an increase of the cell volume, number of branches, and branch length (426 cells in CTL, n_CTL_ = 4; 445 cells in VPA, n_VPA_ = 4, _CTL_ = 4, n_VPA_ = 4). Data analyzed by Shapiro–Wilk and Student’s *t*-test, **p < 0.01, ***p < 0.0001. Values are mean ± SEM.

The expression of GFAP was tested by Western blot with protein samples isolated from cerebella from both experimental groups (CTL and VPA) in 4 independent sets of 4 cerebella each (n_CTL_ = 16, n_VPA_ = 16, each group). Our results showed that the expression of GFAP was increased in the VPA model (28%) compared to CTL group (Figure 4A). Our next step was to test if higher expression of GFAP correlates with a rise in the density of GFAP^+^ cells. Thus, GFAP^+^ cells were counted in the posterior regions of the cerebellum of GFAP-eGFP transgenic mice, Crus I, Crus II and the vermis region of the VII lobule. The density of GFAP^+^ cells increased 260% in the VPA model (18 ± 3 cells, *n* = 4, *p* = 0.005) when compared to CTL (5 ± 1 cells, *n* = 3) (Figures 4B,C). Morphological analysis of individual astrocytes (CTL = 426 cells and VPA = 445 cells; *n* = 4 for each experimental group) showed increased complexity of astrocytes in the VPA model. Thus, the cell volume increased 42% (CTL: 1,188 ± 42 μm^3^, VPA: 1,690 ± 106 μm^3^, *p* = 5.5 × 10^–6^), the number of branches increased 17% (CTL: 5 ± 0.2, VPA: 6 ± 0.2, *p* = 0.009), and the branch length increased 33% (CTL: 29.5 ± 1.2 μm, VPA: 39.3 ± 1.9 μm, *p* = 1.3 × 10^–5^), when compared to CTL group (Figure 4D). We conclude that GFAP expression, astrocyte density and morphological complexity are increased in the VPA model.

## Discussion

Brain plasticity resides in the capabilities of nerve cells to generate and convey signals. Glial cells sense and respond to neuronal activity with intracellular calcium transients that can propagate to neighbor cells in a wave-like fashion (Deitmer et al., 2006; Scemes and Giaume, 2006). In this study we tested the cellular response of the IGL to depolarization in a preclinical model of autism. The cell recruitment, the calcium-wave propagation and the mean amplitude of the recorded calcium transients evoked by depolarization of the IGL were augmented in the VPA model. These functional analyses were performed on mice that showed sensorimotor deficits, increased expression of GFAP, astroglial density and morphological complexity due to prenatal exposure to VPA.

### Sensorimotor Deficits

The increased latencies in righting reflex and nest seeking are in agreement with previous studies using the VPA model (Schneider and Przewłocki, 2005; Roullet et al., 2010; Favre et al., 2013; Kazlauskas et al., 2016; Varman et al., 2018; Wang et al., 2018; Tartaglione et al., 2019). In those studies, autistic subjects were consistently identified based on these neurodevelopmental delays. The sensorimotor delays in the VPA model of ASD correlate with motor deficits observed in ASD children prior to the onset of social or verbal disorders (Lloyd et al., 2013). Experimental data support the examination of early motor deficits as a potential indicator of ASD (Brisson et al., 2012; Sacrey et al., 2015).

### The Functional Signature of the IGL

Neuron-glia communication is required for normal functioning of the brain during early neurodevelopment and throughout life. The intracellular Ca^2+^ transients occur spontaneously or in response to neuronal depolarization, and propagation to neighboring cells results in a calcium wave (Kumada and Komuro, 2004; Hoogland et al., 2009; Verkhratsky et al., 2012; Apuschkin et al., 2013). Thus, we evoked a calcium-wave by depolarizing the IGL and determined the number of recruited cells. Our results showed that >50% of the recruited cells were astrocytes, whereas the other half may correspond to GCs and/or neuronal precursors. In agreement, previous studies showed that calcium waves are ATP-driven and expand passing through glial and GCs of the cerebellum (Hoogland et al., 2009; Apuschkin et al., 2013). Calcium waves are thought to ensure correct wiring of the cerebellar circuits, and the intracellular calcium transients recorded in granular cell precursors are known to be involved with migration through the cerebellar cortex (Kumada and Komuro, 2004; Hoogland et al., 2009; Apuschkin et al., 2013). Regarding the dynamics of the calcium wave, our results showed that the propagation, the cell recruitment, and the mean amplitude of the recorded calcium transients were significantly increased in the VPA model. These results correlate with bioinformatic studies in the ASD context, where calcium signaling associated genes are proposed to be dysregulated in the developing IGL of the cerebellum (Menashe et al., 2013; Zeidán-Chuliá et al., 2013). This is highly relevant considering that GCs represent approximately half of the neurons of the human brain (Wang et al., 2014). GCs receive excitatory synaptic input from mossy fibers and integrate many different sensory receptive fields (Wang et al., 2014). The axons of GCs originate the parallel fibers, a glutamatergic input that projects into the molecular layer (ML) and contact the dendrites of PCs. Parallel fiber synapses begin to appear at P7 in mice (Zwaigenbaum et al., 2013) and normal calcium signaling in the IGL is necessary for the correct wiring of the cerebellum (Kumada and Komuro, 2004; Hoogland et al., 2009; Apuschkin et al., 2013). Our results show that the functional signature of the IGL is augmented and this may correlate with the excessive apoptosis of the GC precursors previously observed in the VPA model (Wang et al., 2018). Parallel fibers are the presynaptic input of PCs and abnormal function of IGL network might explain the impaired dendritic arborizations and synaptic transmission observed in PCs (Wang et al., 2018). Dysfunction of the cerebellar circuitry is involved, in part, with the sensorimotor deficits observed in autism during early postnatal development (Wang et al., 2014).

### Astroglia Is Part of the IGL Functional Signature

Astroglia organizes the architecture of neural networks, nurture synapses and modulate synaptic activity, promoting their development and maturation in the brain (Auld and Robitaille, 2003; Steinmetz et al., 2006; Verkhratsky et al., 2012). The IGL contains cerebellar glomeruli consisting of granule cell dendrites, Golgi cell axon terminals and mossy fibers, all of them wrapped by astrocytes. However, little is known about astroglial integration into the functional network of the IGL. In this study we depolarized the IGL and evoked a calcium wave where half of the recruited cells corresponded to astroglia. This functional signature was augmented in the VPA model with a significant increase in astrocyte recruitment. Accordingly, western blot and histological studies showed an increased expression of GFAP that correlated with an augmented density of astroglia and morphology complexity in the IGL of VPA exposed mice. However, previous studies using the VPA model reported no differences in GFAP-protein expression, but a decreased density of astrocytes was observed in older mice (P35) (Kazlauskas et al., 2016; Bronzuoli et al., 2018). A possible explanation for these discrepancies may be that we used sensorimotor deficits as a prognostic tool to identify autistic individuals which were selected for histological and functional analyses. In support of our results, Western blot studies showed increased expression of GFAP, whereas immunofluorescence studies reported increased density of astrocytes in the cerebellum of *postmortem* brains from ASD patients (Laurence and Fatemi, 2005; Vargas et al., 2005; Edmonson et al., 2014). Thus, augmented density of astrocytes reported in ASD patients may result in a functional response with increased recruitment of this cell type as observed in the VPA model. On the other hand, the mean amplitude of the calcium transient was unchanged in astrocytes. However, homeostatic functions of astroglia include calcium buffering of the glomeruli (Eagleman et al., 2013) and this task may be disturbed by increased density and morphological complexity of this cell type, resulting in an increased neuronal calcium transient amplitude in the VPA model. Augmented calcium signaling is known to delay migration of GC precursors and increased apoptosis of GCs is observed in the VPA model (Kumada and Komuro, 2004; Apuschkin et al., 2013; Wang et al., 2018), consequently, a reduced dendritic arborization of PCs was reported in the VPA model (Wang et al., 2018). Overall, we conclude that the functional signature of the IGL is significantly augmented in the VPA model of autism, which may correlate with neurodevelopmental delays observed in ASD.

## Data Availability Statement

The original contributions presented in the study are included in the article/Supplementary Material, further inquiries can be directed to the corresponding author/s.

## Ethics Statement

The animal study was reviewed and approved by the Institutional Committee on Animal Care and Use of Laboratory Animals of the Institute of Neurobiology, UNAM.

## Author Contributions

MBS-O and DR-H performed the conception and experimental design. MBS-O performed data collection, statistical analysis, animal work, and wrote the manuscript. MBS-O, PR-O, AM-T, and DR-H performed analysis of the data, wrote, and edited the manuscript and provided critical input to the final manuscript. All the authors approved the manuscript.

## Conflict of Interest

The authors declare that the research was conducted in the absence of any commercial or financial relationships that could be construed as a potential conflict of interest.

## Publisher’s Note

All claims expressed in this article are solely those of the authors and do not necessarily represent those of their affiliated organizations, or those of the publisher, the editors and the reviewers. Any product that may be evaluated in this article, or claim that may be made by its manufacturer, is not guaranteed or endorsed by the publisher.

## Dedication

This article is dedicated to the memory of Prof. Ricardo Miledi, a pioneer in Neurobiology who inspired the career of many.

## Supplementary Material

Supplementary Figure 1 Sulforhodamine B (SRB) stains GFP^+^ cells from GFAP-eGFP transgenic mouse. **(A)** SRB signal in Crus II showing the internal granular layer (IGL, red rectangle). Scale bar 100 μm. **(B)** Zoom of IGL showing SRB^+^ and GFAP^+^ cells. Merge of both images shows cellular overlay (white). Scale bar 20 μm. **(C)** Summary of the experiments shows that ∼80% of GFAP^+^ cells are SRB^+^ (*n* = 12, *N* = 3). **(D)** Schematic representation of the experimental setting. **(E)** The calcium wave (Fluo4-AM, green) recruited SRB^+^ cells (overlay in yellow). **(F)** Summary of the experiments shows that > 50% of recruited cells are SRB^+^ (*n* = 8, *N* = 5). Scale bar 100 μm. Stim, stimulus; PL, Purkinje layer; WM, white matter. Statistical tests used were Shapiro–Wilk and Mann–Whitney U.

Supplementary Figure 2 Latency of the Ca^2+^ transients correlate positively with the distance of the cell location. The latency of the individual Ca^2+^ transients (Cells_CTL_ = 618, Cells_VPA_ = 838, *n* = 4 each experimental group) correlate positively with the distance of the cell location in both CTL (*R*^2^ = 0.35) and VPA (*R*^2^ = 0.37). The slopes show no differences between groups (*p* = 0.18). Statistical tests used were Shapiro–Wilk and linear regression.

Supplementary Table 1 Recruited cells.

Supplementary Table 2 Recruitment of SRB^+^ cells.

Supplementary Table 3 Effect of current amplitude on calcium wave propagation.
