# Supplementary figures and images for: A Functional Signature in the Developing Cerebellum: Evidence From a Preclinical Model of Autism
Source: Front Cell Dev Biol. 2021 Sep 3;9:727079. doi: 10.3389/fcell.2021.727079 (PMC8448387; doi:10.3389/fcell.2021.727079)

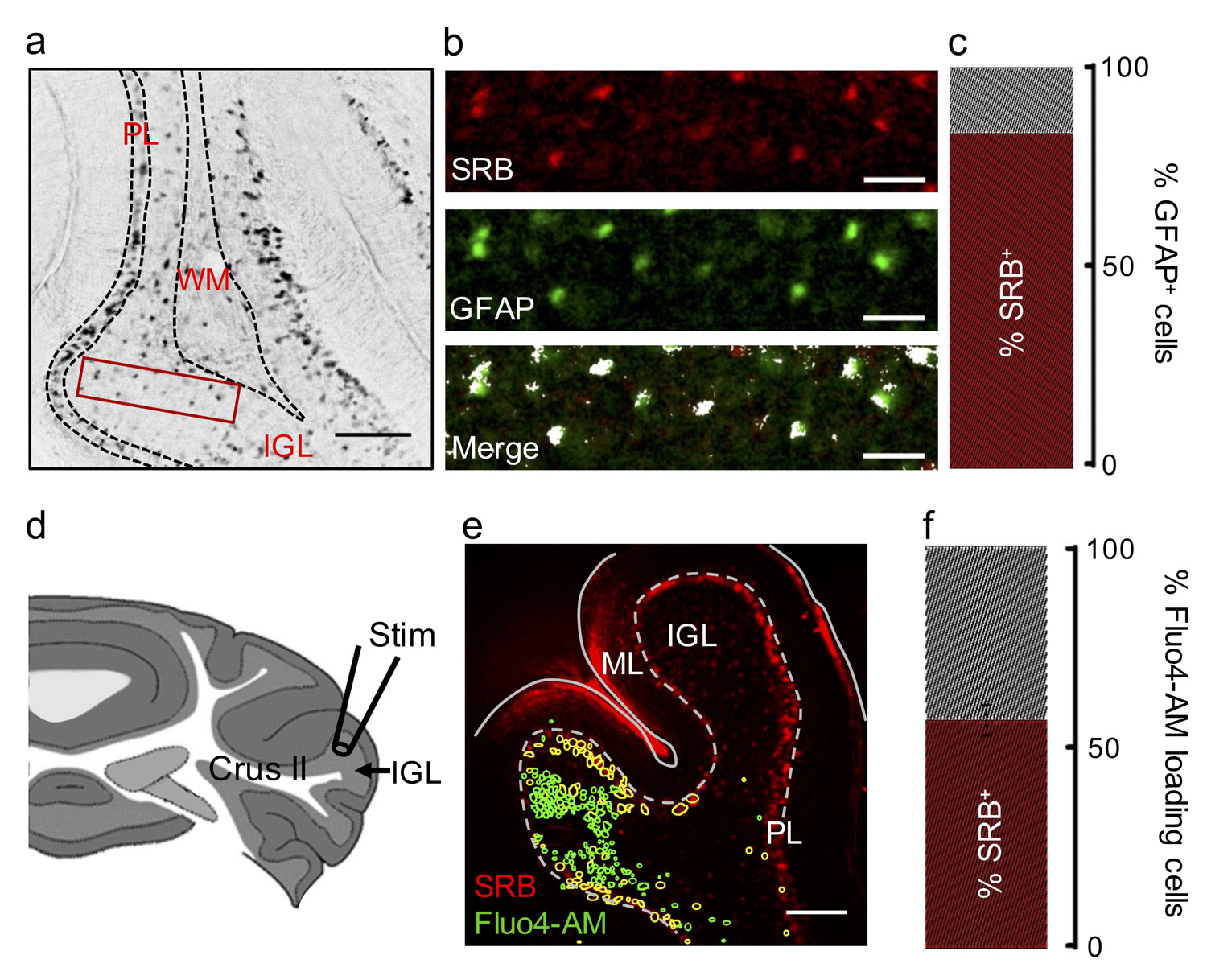

Supplement: Supplementary Figure 1 — Sulforhodamine B (SRB) stains GFP+ cells from GFAP-eGFP transgenic mouse. (A) SRB signal in Crus II showing the internal granular layer (IGL, red rectangle). Scale bar 100 μm. (B) Zoom of IGL showing SRB+ and GFAP+ cells. Merge of both images shows cellular overlay (white). Scale bar 20 μm. (C) Summary of the experiments shows that ∼80% of GFAP+ cells are SRB+ (n = 12, N = 3). (D) Schematic representation of the experimental setting. (E) The calcium wave (Fluo4-AM, green) recruited SRB+ cells (overlay in yellow). (F) Summary of the experiments shows that > 50% of recruited cells are SRB+ (n = 8, N = 5). Scale bar 100 μm. Stim, stimulus; PL, Purkinje layer; WM, white matter. Statistical tests used were Shapiro–Wilk and Mann–Whitney U. [file Image_1.JPEG]

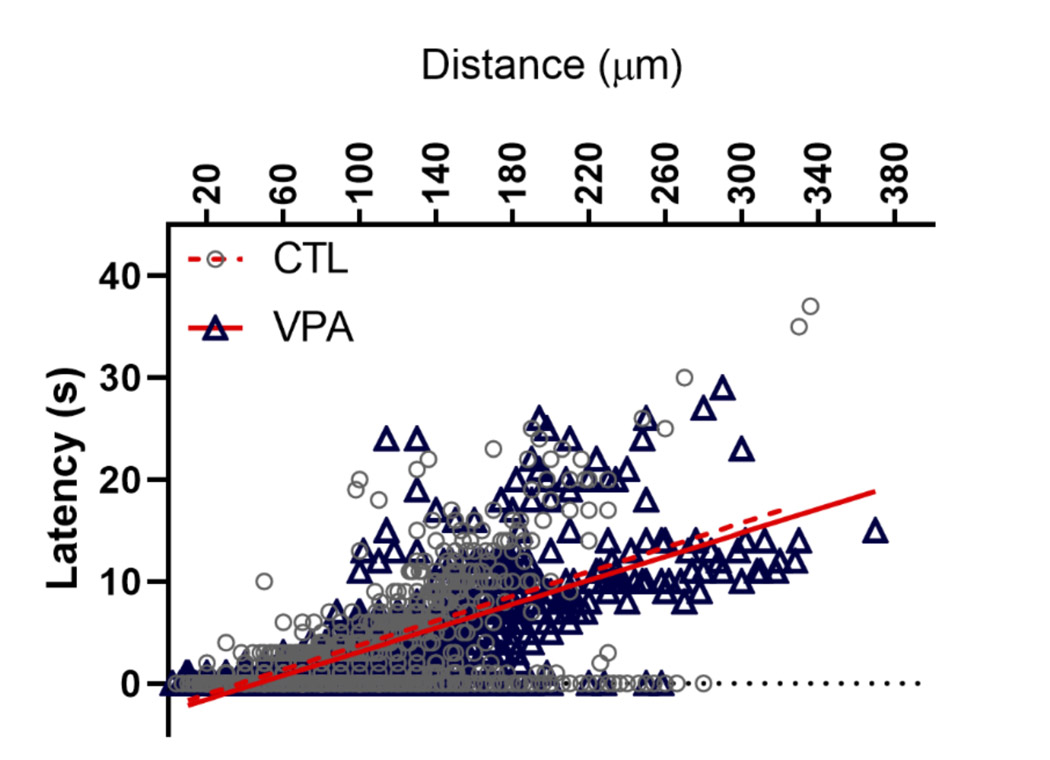

Supplement: Supplementary Figure 2 — Latency of the Ca2+ transients correlate positively with the distance of the cell location. The latency of the individual Ca2+ transients (CellsCTL = 618, CellsVPA = 838, n = 4 each experimental group) correlate positively with the distance of the cell location in both CTL (R2 = 0.35) and VPA (R2 = 0.37). The slopes show no differences between groups (p = 0.18). Statistical tests used were Shapiro–Wilk and linear regression. [file Image_2.JPEG]
