# Supplementary material for: A Functional Signature in the Developing Cerebellum: Evidence From a Preclinical Model of Autism
Source: Front Cell Dev Biol. 2021 Sep 3;9:727079. doi: 10.3389/fcell.2021.727079 (PMC8448387; doi:10.3389/fcell.2021.727079)
Supplement: Supplementary Table 1 — Recruited cells. [file Table_1.DOCX]

Supplementary tables

Table 1. Recruited cells

| Radius  (mm) | CTL  (No. of cells) | VPA  (No. of cells) | *p*-value |
| --- | --- | --- | --- |
| 0-50 | 22 ± 3 | 40 ± 4 | **0.001**^a^** |
| 50-100 | 51 ± 6 | 73 ± 7 | **0.02*** |
| 100-150 | 37 ± 7 | 40 ± 3 | 0.7^a^ |
| 150-200 | 14 ± 4 | 24 ± 5 | 0.14^a^ |
| 200-250 | 4 ± 2 | 15 ± 6 | 0.07 |
| 250-300 | 3 ± 1 | 8 ± 3 | 0.19 |
| 300-350 | 1 ± 1 | 3 ± 1 | 0.14 |
| 350-400 | 0 | 2 ± 1 | **0.03*** |

Data are presented as mean ± SEM. Sample size was n_CTL_= 10,

N_CTL_= 7, n_VPA_= 10, N_VPA_= 9. *p < 0.05, **p < 0.001. Statistical

analysis used was Mann-Whitney. *p < 0.05.

Table 2. Recruitment of SRB^+^ cells

| Radius  (mm) | CTL  (No. of cells) | VPA  (No. of cells) | *p*-value |
| --- | --- | --- | --- |
| 0-50 | 7 ± 1 | 7 ± 2 | 0.83 |
| 50-100 | 17 ± 4 | 20 ± 3 | 0.59 |
| 100-150 | 8 ± 2 | 22 ± 6 | **0.03*** |
| 150-200 | 5 ± 1 | 8 ± 2 | 0.29 |
| 200-250 | 2 ± 0.4 | 3 ± 1 | 0.4 |
| 250-300 | 2 ± 0.6 | 2 ± 0.6 | > 0.9 |
| 300-350 | 3 ± 1 | 1 ± 0 | 0.24 |
| 350-400 | 3 ± 1 | 1 ± 1 | 0.41 |

Data are presented as mean ± SEM. Sample size was n_CTL_ = 6,

n_VPA_ = 4, and N_CTL_ = 4, and N_VPA_ = 3. Statistical analysis used

was Mann-Whitney. *p < 0.05.

Table 3. Effet of current amplitude on calcium wave propagation

| Amplitude  (µA) | Maximum length ± SEM  (mm) | Number of  slices | *p* – value |
| --- | --- | --- | --- |
| 200 | 370 ± 58 | 5 |  |
| 300 | 271 ± 24 | 7 | 0.12 |
| 400 | 240 ± 24 | 5 | 0.17 |
| 500 | 283 ± 60 | 3 | 0.23 |
| 600 | 150 ± 76 | 3 | ***0.02** |

Data are presented as mean ± SEM. Sample size was n_CTL_ = 23 in total.

Statistical analysis used were Shapiro-Wilk and one-way ANOVA followed

by Holm-Sidak’s multiple comparison test.
